# The Use of Smartphone Fitness Applications: The Role of Self-Efficacy and Self-Regulation

**DOI:** 10.3390/ijerph17207639

**Published:** 2020-10-20

**Authors:** Anna Vinnikova, Liangdong Lu, Jiuchang Wei, Guangbao Fang, Jing Yan

**Affiliations:** 1 School of Management, University of Science and Technology of China, Hefei 230026, China; vinnikova@mail.ustc.edu.cn (A.V.); weijc@ustc.edu.cn (J.W.); yj8505@mail.ustc.edu.cn (J.Y.); 2 Faculty of Education, Monash University, Melbourne 3800, Australia; fgb20071507@163.com

**Keywords:** fitness app, UTAUT, SCT, behavioral intention, usage behavior

## Abstract

With the popularity of the health and wellness trend in recent years, smartphone fitness applications have become more and more popular. Thus, this study explored factors affecting the behavioral intention to use and the actual usage behavior of smartphone fitness apps from technical, health, and social perspectives by integrating the Social Cognitive Theory (SCT) and Unified Theory of Acceptance and Use of Technology (UTAUT). We examined whether perceived usefulness, perceived ease-of-use, social influence, self-efficacy, goal-setting, and self-monitoring predict usage behavior. Based on the survey responses of 1066 smartphone fitness apps users, we revealed that all of the variables, except for self-monitoring, significantly influence usage behavior, while behavioral intention acts as a total mediator between perceived usefulness, perceived ease-of-use and usage behavior. Drawing on the research findings, we suggest that influencing behavioral intention to use a fitness app can be an effective method to increase its adoption. Therefore, app developers need to pay attention to interventions that seek to enhance the usefulness of the app, provide professional counseling, as well as an opportunity for effortless goal setting features.

## 1. Introduction

The global smartphone fitness application market has been growing at a rapid pace. Emerging smartphone technology apps offer various capabilities and benefits for consumers and providers of behavioral health care. These apps cover a wide array of areas including diet, exercise, weight loss, nutritional values, and vegetarian choices [1].

In order to combat and prevent the spread of Covid-19, governments across the globe have imposed various movement restrictions and lockdowns. Being isolated may trigger fear and anxiety and cause stress in people. In light of the global pandemic, there will be substantial increases in anxiety, depression, and loneliness [2]. Moreover, self-isolation will negatively affect people’s physical activity levels, and increased screen time will impact physical health, well-being, sleeping patterns, and quality of life. Previous research has established beneficial effects of regular physical activity on many health outcomes [3], including physical and physiological health parameters and positive health outcomes in areas of mental health and well-being [4]. Therefore, home-based activities that smartphone fitness applications offer provide an opportunity for people to stay fit and healthy by practicing movement while staying at home.

Although the download rate of mobile fitness apps is high, so is the uninstall rate. The primary reasons for the users to discontinue using an app are high data entry burden, loss of interest, and hidden costs [5]. Although these apps claim to promote positive lifestyle changes, content analysis of existing apps has identified gaps between evidence-based guidelines and app content relating to weight loss [6,7] and exercise [8]. While some of the previous systematic reviews of using smartphone apps to promote physical activity and weight loss found a non-significant difference in physical activity between the control group and smartphone intervention group [9], others found that smartphone apps can be effective in increasing physical activity [10]. A more recent review suggests that app-based physical activity interventions are the most effective in the short term [11].

To analyze which constructs are significantly related to and potentially impact and/or mediate usage behavior, we employ variables from Social Cognitive Theory (SCT) and Unified Theory of Acceptance and Use of Technology (UTAUT). SCT has been used extensively and successfully to explain, predict, and elicit health behavior change [12], including physical activity [13]. SCT proposes that self-efficacy influences behavioral outcomes in both direct and indirect ways and assumes that behavior change occurs through changes in motivation and self-regulation; health habits are acquired through self-management, which requires motivational and self-regulatory ability and skills [14]. Self-management is summarized in three categories: self-monitoring of behavior, goal-setting to manage efforts and strategies, and social support to maintain health behavior. We believe that to better explain and understand the behavioral intention–usage relationship, different constructs and principles in SCT need to be measured, realized, and manipulated in systematic experiments replicated over various behaviors and populations. A thorough analysis could reveal that some of these concepts and principles are more or less useful or feasible for particular behaviors or types of behavior change. It is essential that we better understand which constructs are significantly related to and potentially impact and/or mediate usage behavior to develop more effective and efficient physical activity interventions for the users. By understanding which psychosocial factors predict users’ behavioral intention and app usage behavior, fitness app developers will be able to create strategies that target these factors. Thus, by using Bandura’s Social Cognitive Theory as a framework with a focus on social cognitive determinants, the present study aims to examine the degree to which self-efficacy, self-regulation, and social influence affect the intention–usage relationship.

To explain the behavior–intention relationship further we used UTAUT. The theory presents four constructs that determine users’ acceptance and usage behavior: performance expectancy, effort expectancy, social influence, and facilitating conditions. The constructs of the UTAUT have been positively associated with the intention to adopt a fitness app [15]. UTAUT overlaps with a general theory that explains individual behavior—the Theory of Reasoned Action (TRA) [16]. According to TRA, behavioral intention is formed by two key variables: attitude and subjective norm. Attitude is “the person’s general feeling of favorableness or unfavorableness toward some stimulus object”; subjective norm, which corresponds to social influence, is the individual’s belief that people whose expectations are perceived to be relevant think he should or should not perform the behavior in question [16]. The two variables in UTAUT, perceived usefulness and perceived ease of use (which correspond to performance expectancy and effort expectancy), are two beliefs resulting in attitude. Behavioral intention in both instances represent the same concept.

## 2. Literature Review

### 2.1. Perceived Usefulness, Perceived Ease-of-Use, Social Influence, and Behavior Intention

Previous studies have shown that technology acceptance is mainly dependent on perceived usefulness and perceived ease-of-use [17,18]. Perceived usefulness is defined as an extent to which an individual believes that the fitness application will assist him or her in managing their weight and healthy diet. Perceived ease-of-use determines the degree of belief that a particular fitness app can be easily used without assistance. Although some studies show that only perceived usefulness predicts intention to use mHealth technology [19], most studies show that both perceived usefulness and perceived ease-of-use have a significant impact on intention to use [20,21]. In the context of smartphone fitness apps, most of the studies focused on initial app adoption [15]. One study that used the TAM framework to predict users’ continuance usage [22] found that perceived usefulness and perceived ease of use, along with social norms, predicted intentions to continue using a particular fitness app among users. It is evident that functionality should be the most prominent quality of fitness apps; hence, our main assumption is that perceived usefulness and perceived ease-of-use affect usage intention and, thus, usage behavior.

Social influence refers to the individual’s perception that one’s important others believe that he or she should use a mobile fitness application to manage one’s diet and weight [23]. While using the original UTAUT model, various scholars have proved social influence to be one of the most critical constructs to explain users’ adoption behavior [24,25,26,27]; others found social influence not to keep to behavioral intent [28]. Drawing on the prior research that has established these relationships, we propose that:

**Hypothesis 1** **(H1).** *Perceived usefulness positively influences (a) behavioral intention and (b) usage behavior*

**Hypothesis 2** **(H2).** *Perceived ease-of-use positively influences (a) behavioral intention and (b) usage behavior*

**Hypothesis 3** **(H3).** *Social influence positively influences (a) behavioral intention and (b) usage behavior*

### 2.2. Self-Efficacy and Behavioral Intention

Self-efficacy [29] in a health context is defined as confidence in one’s ability, knowledge, or skills to engage in a healthful behavior. It is not an indicator of skill, but rather an indicator of belief in one’s skills. When individuals confident in their ability to manage their health are given an opportunity to use a fitness application to monitor their exercise and diet, they are more likely to feel comfortable with the application and perceive it useful. Studies support that self-efficacy has a positive influence on the intention to use mobile services [30] as well as intention to engage in physical activity [31,32]. However, a more recent study suggests that mobile applications with gamification features significantly affect users’ physical activity levels without affecting their self-efficacy [33]. Given that smartphone fitness apps’ primary function is to aid users’ physical activities, self-efficacy is related to people’s adoption and continued use of smartphone fitness apps [34]. However, the role of self-efficacy remains unclear, since little academic attention has been given to this particular context. To the best of our knowledge, in the context of fitness apps, one study found partial evidence for the direct effect of self-efficacy on fitness app usage intentions [34]. Based on the contradicting findings in the previous research, the relationship between self-efficacy and behavioral intention is worth revalidating in the context of mobile fitness applications. Therefore, we hypothesize that:

**Hypothesis 4** **(H4).** *Self-efficacy positively influences (a) behavioral intention and (b) usage behavior*

### 2.3. Self-Efficacy and Self-Regulation

The concept of self-regulation represents how people assess their progress toward reaching their goals and alter their actions accordingly. Studies that explored the relationship between self-regulation and self-efficacy found that individuals who possess higher levels of self-efficacy use self-regulation strategies more frequently [35]. By mediating the relationship between self-efficacy and physical activity, self-regulation facilitates behavior change [36]. Research has consistently suggested that self-efficacy is associated with improved self-regulation; an increase in both is a viable mechanism by which individuals exert their influence on behavior [37]. The impact of self-efficacy on behavior change occurs through self-regulation [14]. Health habits are not changed through willpower alone, but through self-management requiring self-regulatory ability and skills. Hence, we can conclude that self-efficacy might influence physical activity through self-regulation strategies (e.g., thoughts, goals, plans, and acts) that increase physical activity, but this idea has not been widely examined. Two cross-sectional studies that did examine these relationships [36,38] found that although self-efficacy was significantly related to physical activity, self-regulation was determined as a partial mediator of these relationships in both instances. Experiments that combined physical activity training with strategies that facilitate other motivational factors including self-regulation were found to be effective [39]. Better understanding of the relationship between self-efficacy, self-regulation, and physical activity may be helpful in better understanding, predicting, and changing physical activity levels in smartphone fitness users. Therefore, we propose:

**Hypothesis 5** **(H5).** *Self-efficacy has a positive influence on (a) goal-setting and (b) self-monitoring*

### 2.4. Self-Regulation and Usage Behavior

Self-monitoring in fitness apps refers to the “design that allows the user to track his or her performance or status” [40]. Effective self-monitoring includes systematic observation of one’s behavior. It involves observing and recording both the behavior itself and the context and cues or events accompanying the behavior. When combined with goal-setting, self-monitoring has been shown to be an effective component of interventions to promote users’ physical activity [41]. Scholars have found evidence that self-monitoring is significantly affecting adults’ physical activity levels, further suggesting that when technology is able to aid the users in self-monitoring of their performance and progress toward their goals, the level of their commitment, as well as the credibility of the application, increases [42]. On the contrary, an empirical study found that although monitoring and tracking features were found valuable, users expressed that they might prove to be overly burdensome [43].

Researchers recognize goal-setting as a strategy aimed at aiding self-monitoring [44]. Goal setting is a planned behavior that helps us understand the translation of intention into action. Previous studies point out that self-regulatory processes may explain how intentions drive behavior [45], with some authors suggesting that self-regulation is an essential factor in changing peoples’ behavior [46]. Goal-setting, as well as self-monitoring, are necessary to attain behaviors specified by behavioral intentions. A body of research from the user’s perspective has suggested self-monitoring, self-regulation, and goal attainment to be the most desired functions among fitness app users [47,48]; however, the analyses of the prior studies were mainly descriptive in nature and did not systematically link those technological functions with users’ post-adoption behaviors. Therefore, we propose:

**Hypothesis 6** **(H6).** *Goal-setting positively influences (a) behavioral intention and (b) usage behavior*

**Hypothesis 7** **(H7).** *Self-monitoring positively influences usage behavior*

### 2.5. Behavioral Intention and Usage Behavior

Technology acceptance models (TAM, TAM2, and UTAUT) all specify that behavioral intention is the most direct antecedent of technology use, a relationship supported by empirical tests of each model, respectively [17,18,49,50]. A study that examined factors influencing consumers’ intention and actual usage of sports brand apps found that behavioral intention positively affected actual behavior [51]. These examples suggest that behavioral intention has a substantial influence on usage behaviors. Therefore, we propose:

**Hypothesis 8** **(H8).** *Behavioral intention positively influences usage behavior*

**Figure 1:**
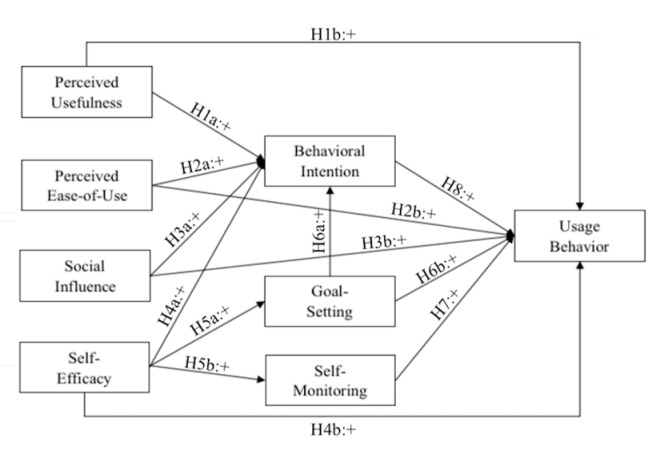
Conceptual framework of understanding and predicting adoption of smartphone fitness apps.

Based on the above analysis, the research framework and model is depicted in Figure 1.

## 3. Materials and Methods

### 3.1. Sampling and Data Collection

To investigate smartphone fitness apps users’ behavioral intention and usage behavior, we conducted a questionnaire survey. Data was collected between 18–25 May 2020, through online survey design and dissemination platform Wenjuanxing [52]. This platform is used by many companies and universities in China to conduct online surveys [53]. It also provides a sampling pool of more than 260 million registered users in China, representing a diverse demographic background. Wenxuanxing randomly contacted its users with brief information about the research and then recorded their responses to the survey.

The first part of the questionnaire briefly introduced the research purpose and thanked the respondents for their participation. It also included consent that was indicated by the individuals to participate in the study voluntary. No identifiable information was collected. Participants could withdraw from the study at any time. The second part comprised questions on the demographic characteristics of the respondents. The final part included scale questions on the research constructs.

Although our questionnaire was in Chinese, the original was developed in English. Therefore, we invited three professional translators to help us translate it into Chinese and back-translate it into English. We compared the different versions of the translation and modified or deleted content that could not be applied in the context of Chinese habits and culture in order to ensure the content validity of our survey. To further check and improve our questionnaire, we used several convenience pretests.

Only the respondents who indicated to be using a fitness app on their smartphones at the time of filling out the questionnaire were included in the study, non-users were excluded. A total of 1066 responses were included in the analysis. Descriptive statistics revealed that males comprised 49.1% (n = 524) and females comprised 50.9% (n = 542) of the participants. The majority of the participants were aged between 18 to 30 (52.3%, n = 558). As for education, most of the participants were undergraduates (70.5%, n = 751), with the majority reporting their annual income as more than ¥100,000 (34.8%, n = 371) (Table 1).

### 3.2. Measures

Perceived usefulness is defined as the extent to which an individual believes that he or she would benefit from using mobile fitness applications. To measure perceived usefulness, we used four items from Davis’s scale [17], which were paraphrased to reflect the use of fitness apps (e.g., I find the fitness app useful in managing my health). The alpha coefficient of internal reliability was 0.908.

Perceived ease-of-use in the context of the present research paper determines how easy the users perceive the fitness application to be. In the context where users have to adopt a fitness application, social influence refers to the degree to which an individual perceives his or her family members, colleagues, friends or medical team to believe they must use the application to manage health and wellness. To measure perceived ease-of-use and social influence, we used three items each, which were adapted from [27,54]. The alpha coefficients of internal reliability for perceived ease-of-use and social influence were 0.869 and 0.909, respectively. 

Self-efficacy in a health context is defined as confidence in one’s ability, knowledge, or skills to engage in a healthful behavior. Three items used to measure self-efficacy were adapted from [55,56]. The alpha coefficient of internal reliability was 0.908.

Behavioral intention was assessed by measuring three items from [57]. The alpha coefficient of internal reliability was 0.833.

We used two dimensions to measure self-regulation: goal-setting [35] and self-monitoring [58]. Goal-setting is a planned behavior in which intentions are formulated regarding both long-term and short-term goals that will bring people closer to the changes they desire. Self-monitoring in fitness apps refers to the design that allows the user to track his or her performance or status. The alpha coefficients of internal reliability for these two items were 0.801 and 0.772, respectively.

The present study used self-reported measures of fitness application usage. Commonly used system components were assessed as recommended by previous researchers [59]. The alpha coefficient of internal reliability for this item was 0.746.

The constructs, its measures, and sources are presented in Table 2.

## 4. Results

Reliability and validity assessment exploratory factor analysis (EFA) and confirmatory factor analysis (CFA) were conducted in SPSS19 and Amos21 respectively to evaluate the construct reliability, validity and unidimensionality of the multi-item measurement scales in our study. As shown in Table 3, all the Cronbach’s alpha and composite reliability values are greater than 0.70, and the average variance extracted (AVE) values are all greater than 0.50, indicating acceptable levels of reliability.

After performing structural equation modeling (SEM) in AMOS 21, an important issue is the criterion for accepting or rejecting a model, which is presented Figure 1. We adopted [60] recommendations that to be acceptable, a model should satisfy the following conditions: (1) the chi-square value divided by the model degrees of freedom (χ^2^/df) should be below 5 and preferably below 3; (2) “the Steiger–Lind root mean square error of approximation (RMSEA) with its 90% confidence interval (90% CI)” [61] and RMSEA values should be lower than 0.08 and preferably 0.06; (3) standardized root mean square residual (SRMR) values below 0.10 are considered to be favorable; and (4) Tacker–Lewis index (TLI) and comparative fit index (CFI) values above 0.90 and preferably above 0.95 indicate a model fit. From the CFA results, the normed χ^2^ (χ^2^ to df, χ^2^ = 629.69, df = 202) is 3.11, and other indicators (TLI = 0.95, CFI = 0.96, GFI = 0.96, RMSEA = 0.039) are also acceptable, indicating that the overall fit of the CFA model is acceptable. Furthermore, we tested the discriminant validity. In Table 4, all the correlations between each pair of constructs are less than the square roots of the AVE values, which supports the discriminant validity.

**Figure 2:**
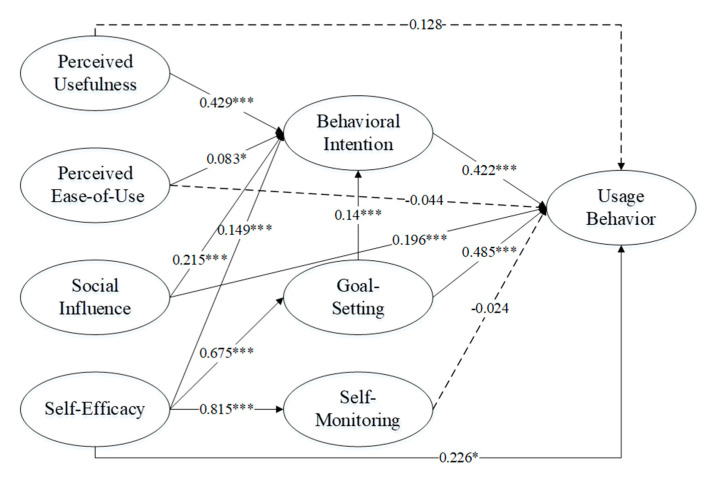
Result of the testing of the model (*** *p* < 0.001; * *p* < 0.05).

First, we measured the direct effect (without any mediation) of independent variables on the dependent. The test of the model provided the estimates of the path coefficients of the direct effects of the model (Figure 2). The results reveal that perceived usefulness (path coefficient of 0.429, *p* < 0.001) and perceived ease-of-use (path coefficient of 0.083, *p* < 0.05) produce a direct effect on behavioral intention, supporting H1a and H2a. Similarly, a path coefficient of 0.251, *p* < 0.001 shows that social influence is correlated with behavioral intention, validating H3a. Furthermore, self-efficacy and behavioral intention were correlated with a path coefficient of 0.675, *p* < 0.001, which supported H4a. It was found that goal-setting produced a direct effect on behavioral intention (path coefficient of 0.140, *p* < 0.001) and usage behavior (path coefficient of 0.485, *p* < 0.001), supporting H6a and H6b. Self-efficacy influenced both goal-setting (path coefficient 0.675, *p* < 0.001) and self-monitoring (path coefficient 0.815, *p* < 0.001). These results supported H5a and H5b. Moreover, social influence (path coefficient of 0.196, *p* < 0.001), self-efficacy (path coefficient of 0.226, *p* < 0.05), and behavioral intention (path coefficient of 0.422, *p* < 0.01) produced a significant direct effect on usage behavior, which supported H3b, H4b, and H8. Contrary to our expectations, perceived usefulness (path coefficient of 0.128, *p* > 0.05), perceived ease-of-use (path coefficient of −0.044, *p* > 0.05), and self-monitoring (path coefficient of −0.024, *p* > 0.05) produce no direct effect on usage behavior. Therefore, H1b, H2b, and H7 are rejected.

Next, we measured indirect effects, that is how the constructs influence usage behavior through behavioral intention. Perceived usefulness, social influence, and self-efficacy have a significant indirect effect on usage behavior. Therefore, it is necessary to test the mediating mechanism. We performed analyses of the mediating roles of behavioral intention and goal-setting through bootstrap using the software package AMOS 21. Compared with other mediation effect test methods, bootstrap does not rely on standard error, which lets us avoid the problem of inconsistent standard error formula. Bootstrap has a high statistical effect [62,63], and it is the most ideal method to test the mediation effect [64,65]. Based on the bootstrapping, using a random sampling method and repeating the extraction process, we took samples repeatedly from our research samples 1000 times. We use the bootstrap confidence interval of indirect effects with bias-correct percentile method to test the mediation effect. As shown in the Figure 2, it can be found that the mediation effects of behavioral intention and goal-setting are significant. 

Then, we use the causal steps approach to test the total mediation effect [66] or partial mediation effect [67]. The causal steps approach revealed that: (1) self-efficacy and social influence have a significant total effect on usage behavior; (2) the direct effect of self-efficacy and social influence on behavioral intention, and the direct effect of self-efficacy on goal-setting, are equally significant; (3) the direct effect of behavioral intention and goal-setting on users’ usage behavior is significant; (4) the direct effect of self-efficacy and social influence on usage behavior is significant; (5) the direct effect of perceived usefulness and perceived ease-of-use on usage behavior is not significant. The first three steps suggest the existence of the mediation effect of behavioral intention and goal-setting, the fourth step indicts that behavioral intention and goal-setting produce partial mediation effect, and the last step indicates that behavioral intention produces total mediation effect between perceived usefulness, perceived ease-of-use and usage behavior.

The total, direct, and indirect effects are presented in Table 5.

We then factored the mediating effect with calculating coefficients and analyzing the proportion of the mediating effect of different variables in the total effect. The indirect effect of self-efficacy and social influence on usage behavior through behavioral intention accounts for 35.8% and 62.4% of the total effect respectively. The indirect effect of self-efficacy on usage behavior through goal-setting accounts for 64.2%.

## 5. Discussion and Implications

### 5.1. Interpretation of Findings

This research investigates the determinants of usage behavior of smartphone fitness applications from technical, health, and social perspectives. Our results offered support for most of the hypotheses and yielded a better understanding of individuals’ behavioral intentions to use the fitness apps and the actual usage behavior.

The present study revealed that perceived usefulness and perceived ease-of-use significantly affect behavioral intention, which in turn affects usage behavior. Users who perceive the app as being more useful exhibit higher intentions to use this app. Our findings echo the previous finding of a separate survey study based on the TAM framework conducted across university students, which found that perceived usefulness predicts intention-to-use [68], as well as findings of another research, where perceived ease-of-use was a significant factor for medical wearable device users to adopt a wearable medical device [69]. These two variables, however, were found to produce no direct effect on usage behavior. This finding is in line with the findings of another study on fitness apps in China, which found a negative correlation between performance expectancy and usage behavior [70]. Our findings suggest that influencing behavioral intention to use a fitness app can be an effective method to increase its adoption.

Social influence and self-efficacy have an effect on usage behavior through behavioral intention, where behavioral intention acts as a partial mediator. There are over 70 million monthly active fitness app users in China, most of whom are 25–34 years old and grew up in a culture of social sharing. When users observe actions and attitudes among them on a regular basis, others’ opinions become important to them, and it indirectly affects their judgment and influences their behavior. Although some studies found that social influence did not influence users’ intention-to-use mHealth apps [71], more recent research, however, revealed that social influence was one of the key variables that significantly affected individual’s intention to exercise [72] and use of mHealth technology [73]. Moreover, fitness information sharing on social media can lead to friendly competition that might initiate behavior change and improve well-being [74]. Thus, we can conclude that intentions to use fitness apps are shaped by social influence and our expectations of performance and effort.

We have found that users with higher levels of self-efficacy exhibit higher levels of self-regulation (goal-setting and self-monitoring). Our findings are in line with the core idea of SCT: individuals with higher self-efficacy are more likely to implement effective self-regulatory strategies in adopting and maintaining enhanced physical activity behaviors [75]. Perceived self-efficacy pertains to personal action control or agency. In adopting the desired behavior, individuals first form a goal and then attempt to execute the action. Goals serve as self-incentives and guides to health behaviors. Self-efficacy beliefs affect behaviors indirectly through their impact on goals.

While self-efficacy increases usage behavior through goal-setting, contrary to our expectations, it fails to boost usage through self-monitoring. This finding contradicts a study that found self-monitoring to be one of the most desired features among fitness app users [48]. We attribute our finding to the following reasons. First, some users might find it difficult to interpret the self-monitoring results and not know how to translate them into further exercise goals. Previously, diabetes patients expressed frustrations of keeping detailed records of blood glucose, physical activity, or food intake [76]. Ongoing counseling aimed particularly at physical activity has been found to foster long-term improvement [77]. Online counseling can boost effective engagement with fitness applications. Professional online support that enables remote contact with a healthcare professional has been found to positively influence engagement with mHealth technology [78]. It may be useful when the users feel the need for a healthcare professional to reassure, guide, and emotionally support them. By maintaining surveillance of the user’s interactions with the app, expert human facilitation can also lead to greater motivation and an obligation to use the app [79].

To improve users’ experience, fitness apps’ developers could provide personal online consultations with recommendations and further guidelines based on the users’ self-monitoring records. Moreover, the kind of self-monitoring feature that the app provides could explain self-monitoring effectiveness. Research revealed that despite finding journaling helpful, many participants said that they doubted they would have kept up with journaling without reminders [80]. Lastly, some apps do not sync with other fitness devices, and smartphone sensors alone may not be as accurate as other standalone sensor devices. Apps need to keep up with the current developments of smartphone and wireless devices sensor technology to provide more accurate data [74].

### 5.2. Contributions and Implications

Taking the widespread availability of mobile devices among consumers into consideration, they are an excellent way to influence health behaviors. A fundamental question that faces developers of fitness apps is that once consumers download the app, how do you get them to sustain using the app over a long period of time? Findings from such research will not only assist the thousands of fitness app developers but also lend support to the government’s focus on empowering and involving consumers in their health management.

The major theoretical contributions of the present study lie in the following aspects. The present study is one of a few attempts to extend the scope of technology adoption perspective into the context of the continued and actual use of smartphone fitness apps. As previous research on health apps has primarily focused on initial intention to adopt the app [81], few studies have focused on post-adoption behavior [22]. Post-adoption behavior of people using smartphone fitness apps is still a new phenomenon; very little is known about it. Previously, scholars indicated that behavioral aspects and social meanings associated with using a new technology continuously change over time [82]. Therefore, it is necessary to examine smartphone fitness app users’ post-adoption behaviors. To fill this gap in smartphone fitness apps, this study has developed and tested a model to determine psychological and functional factors that affect continuance intention to use smartphone fitness apps and actual usage behavior, theoretically relying on unified theory of acceptance and use of technology (UTAUT) and social cognitive theory (SCT). This study’s main findings contribute to explaining the basic process of motivating smartphone fitness app users to continue using fitness apps. The findings are useful for researches to examine fitness-related technologies further.

Specifically, the current study enriches our understanding of fitness app post-adoption behavior in the following ways. First of all, while UTAUT predictors focus on the users’ generic feelings (usefulness, ease-of-use), they provide insufficient information for fitness app developers and designers as to which specific functions or features are needed in technology adoption. To fill this gap, the present study extends UTAUT from human–computer interaction perspective and examines which technological functions would influence users’ continuance intention and actual use. To the best of our knowledge, this study is the first to link self-regulatory functions with smartphone fitness app users’ post-adoption behavior, as previous studies focused on social functions [72,83]. Besides, we identified moderators (behavioral intention and self-regulatory functions) that explain the impact of the fitness apps’ psychological and technological functions on actual usage behavior.

Moreover, by focusing on smartphone fitness app use in China, this study extends previous studies’ findings to a broader context. China has witnessed exponential growth in fitness and sport, with an estimated value of the Chinese fitness app market exceeding 170 billion USD in 2019 [84]. China has been ranked as one of the top countries in terms of smartphone ownership, with 882.23 million owners in 2019 [85]. Accordingly, people’s app use has continued to increase, with 165 million fitness app users in 2019 [84]. This study will provide a deeper understanding of smartphone fitness apps’ use patterns in more IT-advanced infrastructures.

Under the Transtheoretical Model of Behavior Change [86], respondents participating in the present research can be described as a group in the “action” or “maintenance” stage. Individuals go through different stages of behavior change, and each change is characterized by a certain process. First of all, data analysis results confirm that perceived usefulness of a fitness app influences behavioral intention. The users who go through “action” and “maintenance” stages experience counterconditioning. During this process, users learn about healthier behaviors that can be alternatives to problem behaviors. Users who face a choice between sedentary or active lifestyles may choose the latter provided that the fitness app is perceived as useful. Perceived usefulness enhances users’ engagement in performing healthful behavior and using a fitness app. Perceived effectiveness, on the other hand, is an outcome of usage behavior. After the users engage with the fitness application over a certain period of time, they may perceive it as effective in helping them achieve healthful behavior. Future research should further distinguish between perceived usefulness and perceived effectiveness as constructs and investigate their influence on intention to use and actual usage behavior.

Second, this research shows that social influence is an important predictor of adoption intentions. According to TTM, social support is a key factor to the behavioral change in individuals who are at the “maintenance” stage. One thing that fitness app firms could do is attempt to leverage social networks to induce social contagion [87] of app adoption across the network. For example, app users liking the app on their social media page or spreading the benefits of using the app online could serve to more strongly appeal to the attention of the members in the local member’s social circle. This way, users can find people who support their behavioral change. App developers could also set up professional counseling that provides care, acceptance as well as encouragement to engage in physical activity with the aid of a fitness application.

Third, respondents used in this research are all active users who have maintained regular physical activities with the help of a fitness app. People who have used an app over a period of time are confident of their ability to change, and they commit to that belief. They commit and re-commit on that belief. One of the features that app developers could provide is giving the users an opportunity to set their goals and share their goals with other users. This can enhance the users’ willpower and boost their usage behavior.

Just as with any empirical research, limitations do exist in this study. First, our survey participants are mostly young people, so we must acknowledge the fact that the users’ attitudes and factors that drive their usage behavior may vary across different age groups. Therefore, the generalization of the results may be constrained, and future endeavors remain necessary. Moreover, future studies should further investigate kinds of self-monitoring techniques that are the most beneficial and appealing to app users.

## 6. Conclusions

Unlike previous research on the adoption of fitness apps, this study has investigated individuals’ post-adoption behaviors using smartphone fitness apps. Relying on the unified theory of acceptance and use of technology (UTAUT) and social cognitive theory (SCT), this study examined technological functions by analyzing the data from adult Chinese users of smartphone fitness apps. Moreover, self-regulatory functions in smartphone fitness apps were incorporated to study fitness app users’ post-adoption behavior. The results suggest that behavioral intention and goal-setting produce a partial mediation effect between social influence, self-efficacy, and usage behavior. Perceived usefulness and perceived ease-of-use produce no direct effect on usage behavior. In this case, behavioral intention acts as a total mediator between perceived usefulness, perceived ease-of-use, and usage behavior. These results should be considered by government bodies and fitness app developers to understand the post-adoption behaviors of fitness apps as they are a useful tool for health management.

## Figures and Tables

**Table 1:** Demographic statistics of the respondents (n = 1066).

Variable		Number	Percentage (%)
Gender	Male	524	49.1
	Female	542	50.9
Age	Less than 18	24	2.3
	18–30	558	52.3
	31–40	358	33.6
	41–50	110	10.3
	51–60	16	1.5
Education	Elementary or junior high school	154	14.4
	Undergraduate	751	70.5
	Graduate school and above	161	15.1
Annual income	Less than ¥30,000	241	22.6
	¥30,000–¥60,000	165	15.5
	¥60,001–¥100,000	289	27.1
	More than ¥100,000	371	34.8

**Table 2:** Constructs and items included in the questionnaire.

Construct	Item	Measurement	Source
Perceived Usefulness (PU)	PU1	I find the fitness app useful in managing my health.	[17]
	PU2	Using the fitness app would enhance my effectiveness in managing my health.	
	PU3	Using the fitness app would help me accomplish my health management goals.	
	PU4	Using the fitness app would improve my performance in my health management.	
Perceived Ease-of-Use (PEOU)	PEOU1	Learning how to use the fitness app is easy for me.	[28]
	PEOU2	I find this fitness app easy to use.	
	PEOU3	It is easy for me to become skillful at using the fitness app.	
Social Influence (SI)	SI1	People who are important to me would think that I should use this fitness app.	[55]
	SI2	People who influence me would think that I should use this fitness app.	
	SI3	People whose opinions are valued to me would prefer that I should use this fitness app.	
Self-efficacy (SE)	SE1	It is easy for me to use this fitness app.	[56,57]
	SE2	I have the capability to use this fitness app.	
	SE3	I am able to use this fitness app without much effort.	
Behavioral Intention (BI)	BI1	I intend to use this fitness app in the future.	[58]
	BI2	I intend to use this fitness app at every opportunity in the future.	
	BI3	I plan to increase my use of this fitness app in the future.	
Goal-setting (GS)	GS1	I set short term goals for how often I am active.	[36]
	GS2	I set PA goals that focus on my health.	
Self-monitoring (SM)	SM1	I watch for signs of progress as I stay physically active.	[59]
	SM2	I monitor myself to see if I am meeting my goals for physical activity.	
Usage Behavior (UB)	Frequency	How many times a week do you use fitness apps?	[60]
	Usage duration	How long have you been using fitness apps?	
	Number of apps	How many fitness apps have you used?	

**Table 3:** Confirmatory factor analysis results for measurement model.

Construct	Items	Loadings	Cronbach’s α	Composite Reliability	Average Variance Extracted
Perceived Usefulness	PU1	0.865	0.911	0.933	0.779
	PU2	0.908			
	PU3	0.923			
	PU4	0.833			
Perceived Ease-of-Use	PEOU1	0.914	0.842	0.889	0.729
	PEOU2	0.835			
	PEOU3	0.810			
Social Influence	SI1	0.901	0.909	0.933	0.844
	SI2	0.923			
	SI3	0.899			
Self-efficacy	SE1	0.892	0.868	0.936	0.830
	SE2	0.921			
	SE3	0.920			
Behavioral Intention	BI1	0.877	0.853	0.912	0.776
	BI2	0.853			
	BI3	0.912			
Goal-setting	GS1	0.931	0.821	0.919	0.850
	GS2	0.913			
Self-monitoring	SM1	0.867	0.722	0.875	0.777
	SM2	0.896			
Usage Behavior	UB1	0.701	0.805	0.801	0.575
	UB2	0.803			
	UB3	0.767			

**Table 4:** Means, standard deviation, and correlations.

Item	Mean	SD	UB	PU	PEOU	SI	SE	BI	GS	SM
UB	2.01	0.75	0.575							
PU	3.97	0.69	0.24 **	0.779						
PEOU	4.26	0.75	0.16 **	0.47 **	0.729					
SI	3.67	0.88	0.23 **	0.48 **	0.17 **	0.844				
SE	4.33	0.69	0.22 **	0.48 **	0.69 **	0.21 **	0.830			
BI	3.99	0.73	0.21 **	0.61 **	0.42 **	0.49 **	0.44 **	0.776		
GS	3.81	0.84	0.15 **	0.51 **	0.32 **	0.33 **	0.36 **	0.44 **	0.850	
SM	4.04	0.78	0.19 **	0.50 **	0.42 **	0.27 **	0.42 **	0.46 **	0.50 **	0.777

Note: Correlations appear below the diagonal; the square roots of AVE values appear on the diagonal and present in bold type. ** *p* < 0.001.

**Table 5:** Total effect, direct effect, and indirect effect.

	Total Effects		Direct Effects		Indirect Effects	
	Coefficient Values	Bootstrap S.E.	Coefficient Values	Bootstrap S.E.	Coefficient Values	Bootstrap S.E.
PEOU to UB	0.13	0.141	−0.044	0.051	0.174 *	0.09
PU to UB	0.012	0.1	0.128	0.089	−0.116	0.069
SE to UB	0.397 *	0.144	0.226 *	0.087	0.171 **	0.107
SI to UB	0.337 **	0.069	0.196 *	0.047	0.141 *	0.068
GS to UB	0.604 **	0.087	0.485 **	0.035	0.119 *	0.061
BI to UB	0.422 *	0.223	0.422 **	0.223		
SM to UB	−0.024	0.092	−0.024	0.092		
PEOU to BI	0.083 *	0.037	0.083 *	0.037		
PU to BI	0.429 ***	0.051	0.429 ***	0.051		
SE to BI	0.379 **	0.103	0.149 *	0.573	0.230 **	0.042
SI to BI	0.251 ***	0.027	0.251 ***	0.027		
GS to BI	0.140 ***	0.050	0.140 ***	0.050		
SE to GS	0.675 ***	0.061	0.675 ***	0.061		

*** *p* < 0.001, ** *p* < 0.01; * *p* < 0.05.
